# Faculty Development Workshop for Endoscopic Teaching Techniques

**DOI:** 10.15766/mep_2374-8265.10960

**Published:** 2020-09-23

**Authors:** Kamron Pourmand, Satish Nagula, Laurie Keefer, Brijen Shah

**Affiliations:** 1 Assistant Professor, Department of Medicine, Division of Liver Diseases, Icahn School of Medicine at Mount Sinai; 2 Associate Professor, Department of Medicine, Division of Gastroenterology, Icahn School of Medicine at Mount Sinai; 3 Professor, Department of Medicine, Division of Gastroenterology, Icahn School of Medicine at Mount Sinai

**Keywords:** Endoscopy, Faculty Development, Gastroenterology, Physician, Clinical/Procedural Skills Training

## Abstract

**Introduction:**

Given the substantial variability in endoscopy teaching that gastroenterology fellows can be expected to encounter over the course of their fellowship, it is important to identify a standard set of behaviors that faculty can adopt to help trainees gain competence in endoscopy at an efficient rate. There remains a scarcity of easily distributed, effective tools for faculty development in regard to teaching endoscopy.

**Methods:**

Based on a prior qualitative study, we developed a three-part trigger video to be used for discussion during a faculty development session. We utilized three role-play scenarios between a fellow and supervisor managing a gastrointestinal bleed in the endoscopy suite. We summarized the implementation and instructions in a faculty guide. We used a postsession survey to measure effectiveness of the faculty development session.

**Results:**

This workshop has been successfully administered twice in 2018 with over 30 gastroenterologists and hepatologists involved in endoscopic teaching. Overall, all faculty have found the workshop useful in learning about endoscopic teaching behaviors and helpful in adjusting their own behaviors in endoscopic teaching.

**Discussion:**

We developed a faculty development workshop specifically for teaching endoscopy to gastroenterology trainees that is widely generalizable to other programs. Overall, we found a high level of satisfaction amongst the participants who have completed it. The tools we created can be easily tailored to complement any existing faculty development session and extrapolated to similar procedural disciplines.

## Educational Objectives

By the end of this activity, learners will be able to:

1. Examine the impact positive and negative teaching behaviors in endoscopy have on the learning environment.
2. Apply effective teaching behaviors in endoscopy.
3. Assess one's own endoscopic teaching ability.

## Introduction

Learning to perform endoscopic procedures is a crucial and formative component of gastroenterology training. These procedures have greater cognitive and psycho-motor components than the bedside procedures that new fellows would have performed as internal medicine residents. However, there is an absence of easily accessible tools for faculty development to teach endoscopy. Though there are surgical curricula related to endoscopic teaching,^1^ the context is very different and often the target learner is the trainee. With such variability in the number and quality of preceptors that gastroenterology fellows can be expected to encounter during the course of their fellowship, it is of importance to identify a standard set of behaviors that faculty can adopt to help trainees gain competence in endoscopy at an efficient rate. Faculty development is now a common program requirement as dictated by the ACGME^2^ and equipping faculty with skills to safely teach procedures is a mandate for program leadership.

The knowledge gap in our understanding of which behaviors exist in the endoscopy teaching encounter was the cornerstone of our qualitative analysis.^3^ In this study, we characterized endoscopic teaching behaviors perceived as beneficial and detrimental to learning from the perspective of fellows. Our results included 239 teaching behaviors identified by 19 fellows who worked with 31 supervising physicians. Twenty-nine unique behaviors were identified and organized into seven themes: teaching, learning environment, autonomy, communication, coaching, feedback, and professionalism.

This next phase of our educational project was to utilize these identified behaviors to develop an instructional video for gastroenterologists who are involved with fellowship training programs during a faculty development session. This submission will help to address a national gap in endoscopic teaching resources, which will be generalizable to gastroenterology programs nationwide. This is the first faculty development tool within *MedEdPORTAL* to serve as a resource for endoscopic teaching.

## Methods

### Target Learners

Our product was aimed at gastroenterology and hepatology faculty who were involved in the teaching and supervision of upper and lower endoscopy. Currently, our program does not have formal ongoing faculty development on this topic. Less than 10% of the target audience were advanced endoscopists who taught in national or regional courses where they were instructing others in endoscopy or attending courses on how to teach endoscopy. Approximately a quarter of the group attended a 45-minute faculty development session on endoscopic teaching over 5 years prior to these sessions.

### Instructional Design of the Session

The workshop was designed as a 60-minute live session in a large-group format with one facilitator.

### Development of the Trigger Video

In order to tailor our faculty development tool on endoscopic teaching, a needs assessment was administered to gastroenterology fellows and supervisors to identify which teaching behaviors were most impactful during endoscopy and how often certain behaviors occurred. This needs assessment was informed by our prior qualitative analysis of endoscopic teaching behaviors.^3^ Our needs assessment was administered to gastroenterology trainees and teaching faculty at our institution. Fifty-four participants responded (61.3%), including 42 faculty and 12 trainees. Both trainees and supervisors deemed all 26 behaviors as having at least some learning impact. The four behaviors with the highest concordant Likert scores were chosen to be highlighted in the videos (i.e., demonstrating unfamiliar techniques, discussing endoscopic findings in real time, demonstrating patience, and creating a supportive learning environment).

With regards to supervisor teaching behavior frequency, faculty perceived they performed teaching behaviors more frequently than fellows perceived. In five of these behaviors, the average score between supervisors and trainees was discrepant at least 1.2 points on the 5-point Likert scale. No behaviors were discrepant more than 2 points. The most discrepant teaching behaviors were also emphasized in the videos (including active supervision and providing formal feedback).

Ultimately, the needs assessment helped us clarify that all the behaviors we identified from our previous study had at least some perceived impact on endoscopic learning. It also helped us understand that despite the slight trend that supervisors thought they performed these behaviors more frequently than trainees perceived, there was an overall high level of agreement for which behaviors were important and how often they were performed between both groups. The behaviors with the highest impact scores or biggest discrepancies in performance were chosen to be highlighted in our workshop.

We thought the best tool to demonstrate effective teaching techniques was a taped role-play demonstration, allowing for easy dissemination and consistency. The tool was a scripted role-play of three scenarios including a gastroenterology trainee and supervising attending managing an upper gastrointestinal bleed in the endoscopy suite (Appendices A, B, and C):

• Appendix A: Fellow and attending performed hemostasis of bleeding gastric ulcer. Many negative teaching behaviors, fewer positive behaviors.
• Appendix B: Fellow and attending performed hemostasis of bleeding gastric ulcer. Many positive teaching behaviors, fewer negative behaviors.
• Appendix C: Attending gave the fellow feedback on their completed endoscopy report.

The script was created for each scenario and behaviors from our needs assessment were mapped according to positive and negative valence. This was then incorporated into a faculty guide (Appendix D), so that any gastroenterologist with an interest in endoscopic teaching would be able to implement this faculty development workshop. Video production was supported through microgrant funding from the American Gastroenterological Association's Academy of Educators.

### Required Equipment and Set-Up

A projection screen to display workshop slides and videos was needed, including the printed instructional guide, and AV with sound capability. A whiteboard and markers to write down comments was optional.

### Implementation and Assessment of the Workshop

The facilitator utilized projected slides (Appendix E) and video clips to deliver the content alongside a guide that provided instructions on transition, discussion points, and highlighted teaching behaviors demonstrated in the video clips.

The faculty development workshop was first piloted within a single gastroenterology division (*n* = 15) in our health system. A postworkshop survey was administered after the pilot. This initial workshop allowed for the facilitator to troubleshoot any time constraints and guided discussion. Subsequently, the workshop was administered by the same facilitator to a multidivisional cohort of faculty (*n* = 28) within our health system. A pre- and postsurvey was administered to faculty (Appendix F) to measure effectiveness after the session. These surveys included a 5-point Likert scale to assess various self-reported skills and attitudes on endoscopy teaching and impact that the workshop had on them.

## Results

Our first faculty development session was administered to 15 faculty members within a single academic gastroenterology division, six of whom completed an anonymous survey afterward for evaluation. The facilitator was a gastroenterology faculty member within the same division with expertise in advanced endoscopy. We had a 40% completion rate of our postsession evaluation. All (6/6) faculty thought the session met its learning objectives and that it was interactive. All faculty stated that they were *likely* or *very likely* to change their teaching practices as a result of the session (Table). Comments on self-intended behavioral change included “more clear interactions,” “look at fellow positioning more,” and “giving time for feedback.”

**Table.**
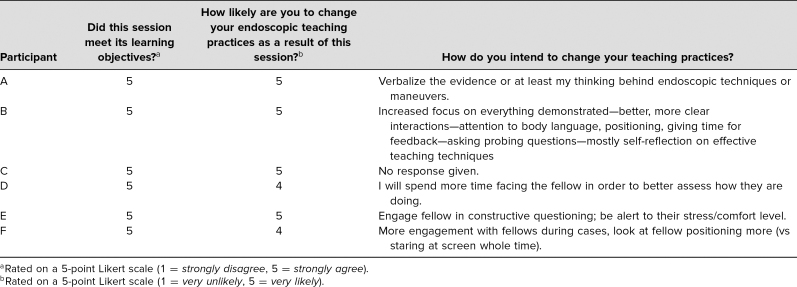
Pilot Workshop Postsession Assessment

**Figure 1.**
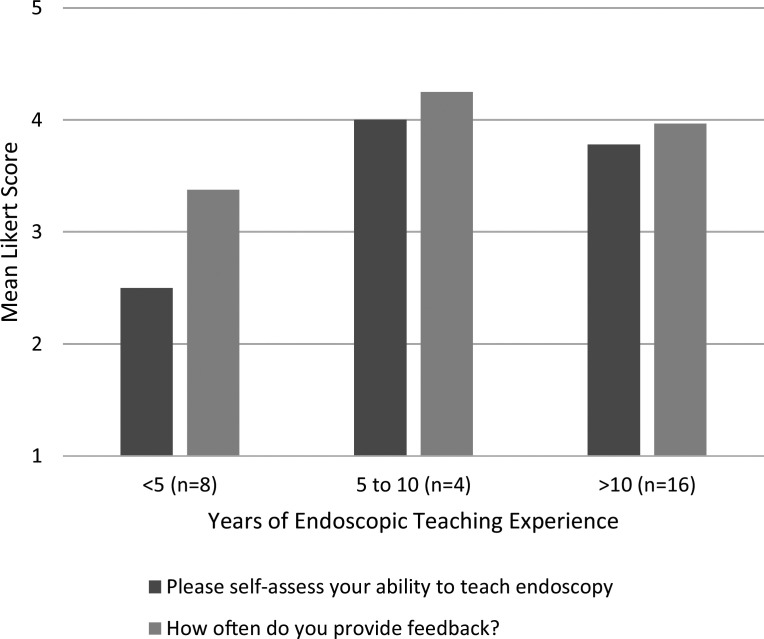
Preworkshop self-reflection mean Likert scores by years of endoscopic teaching experience.

**Figure 2.**
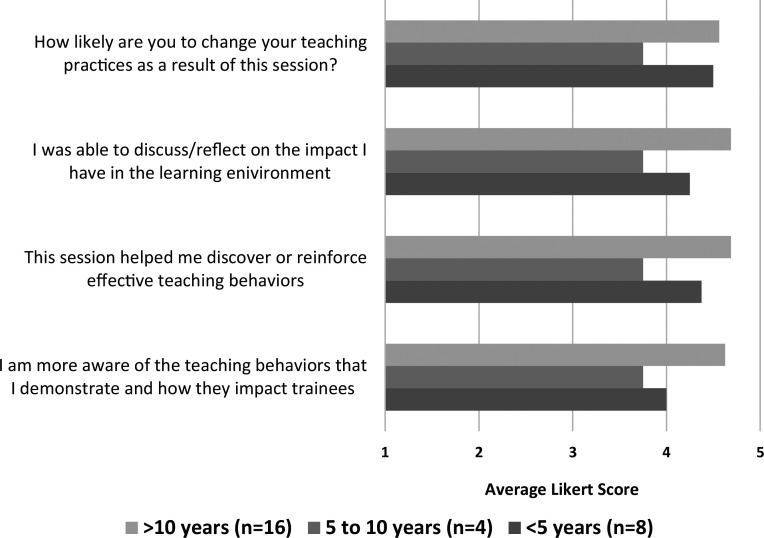
Postworkshop self-reflection mean Likert scores by years of endoscopic teaching experience.

After our second faculty development session, 28 faculty members from three gastroenterology training divisions (two of these divisions were new) attended, including both gastroenterologists and hepatologists. The facilitator was the same person as the initial session. Results were stratified based on years of endoscopy teaching experience. Overall, all faculty felt that the workshop made them more aware of teaching behaviors that exist in endoscopy, helped them reinforce effective behaviors, and thought the workshop would likely change their teaching practices. Pre- and postintervention survey results were listed (Figure 1 and 2 respectively), as well as a sampling of comments from 14 faculty who responded on self-intended behavior change. Responses to, “How do you intend to change your teaching practices?” were as follows:

• “Do a better job going over the endo note with the fellow.”
• “Explaining techniques in the moment.”
• “More feedback.”
• “Much more focused efforts for feedback and hands on instruction.”
• “Verbal feedback postprocedure.”
• “Body positioning.”
• “More discussion between both preceptor and trainee.”
• “More regular feedback after procedures.”
• “Give consistent and timely postprocedure feedback.”
• “Setting goals pre/postprocedure.”
• “More feedback postprocedure.”
• “Provide more formal feedback.”

## Discussion

Derived from learning input, we have developed the first faculty development resource for endoscopic teaching which requires few resources. The strength of this workshop was its ability to be disseminated and incorporated to training programs at different institutions. Utilizing a detailed facilitator guide, any teacher of endoscopy who is interested in endoscopic teaching at a training program can use our scripted videos to lead the faculty development workshop. It can be easily tailored to complement any existing faculty development session. This workshop could also be incorporated into other procedural fields that that utilize endoscopy including interventional pulmonology, urology, ENT, or general surgery.

The development of this faculty resource was iterative and utilized results of our initial qualitative analysis on endoscopic teaching behaviors.^3^ Its implementation was also iterative, with an initial pilot amongst a few gastroenterologists and then administered to a larger, more heterogeneous group of practicing endoscopists. The initial administration of the workshop helped us determine the amount of time that was needed to cover the learning objectives, and second workshop ensured that this time requirement was constant with a larger group. Overall, we found a high level of satisfaction amongst the participants in the workshop's ability to satisfy its learning objectives.

The lessons we learned from the development and implementation of this resource were valuable. We gained an appreciation for the several steps involved in leading educational change at our institution.^4^ We were able to establish a sense of urgency utilizing formal needs assessments and build a guiding coalition with like-minded educators. It was apparent that faculty development projects require buy-in from the divisional leaders to create a shared vision and remove barriers to implementation. As an example, one of our challenges included finding time for several busy faculty members to be present for the intervention. Because our division was able to give us preexisting conference time to pilot the project, we achieved high attendance. Our division chief amplified endorsed the initiative to further increase attendance.

We also underestimated the amount of time needed to foster discussion and share ideas after the trigger videos during our initial pilot session. We were able to rectify this by cutting out some of the didactic portion and allowing more time for discussion in the second iteration of the workshop, which felt much more comfortable in regard to time management.

Another challenge was financial support for video recording and editing. Fortunately, another lesson we learned was that medical subspecialty societies often have resources to support investigations in education. We were able to apply for an educational microgrant through the American Gastroenterological Association to fund the development of the project. This also allowed us an opportunity to connect with other educators and obtain constructive feedback on the project.

One limitation of our resource was that it is operator-dependent, and that it required the facilitator to be familiar with the facilitator's guide and trigger videos so that they can associate participant discussion to key teaching behaviors. However, this afforded creativity for individual facilitators in what aspects endoscopic teaching they would like to focus on and how they would like to deliver it. Our materials should be viewed as a resource for interested and highly motivated gastroenterologists, to help change the knowledge and attitudes of their division as it relates to endoscopic teaching. Another limitation is the fact that this intervention was conducted at a single site, and may not be representative of the unique hurdles at another academic center. The data collected was also self-reported, and we do not yet have data on if teaching behaviors have actually changed or if fellows have improved their endoscopic competency as a result of the intervention. We also do not have any data on the impact of the fellows’ perception of the learning encounters postintervention.

Future directions for this workshop include more rigorous levels of evaluation for its efficacy. We did not objectively measure any change in teaching quality such as from the perspective of the learner postintervention. Ultimately, our goal is improved learner competency in various endoscopic realms as a result of this workshop.

## Appendices

Video 1.mp4Video 2.mp4Video 3.mp4Facilitator Guide.docxWorkshop Slides.pptxPre- and Postworkshop Survey.docx All appendices are peer reviewed as integral parts of the Original Publication.
