# Supplementary material for: Faculty Development Workshop for Endoscopic Teaching Techniques
Source: MedEdPORTAL. 2020 Sep 23;16:10960. doi: 10.15766/mep_2374-8265.10960 (PMC7511062; doi:10.15766/mep_2374-8265.10960)
Supplement: Supplementary file 1 — Video 1.mp4Video 2.mp4Video 3.mp4Facilitator Guide.docxWorkshop Slides.pptxPre- and Postworkshop Survey.docx [file mep_2374-8265.10960-s001.zip › D. Facilitator Guide.docx]

**FACULTY DEVELOPMENT WORKSHOP FOR ENDOSCOPIC TEACHING TECHNIQUES**

**Faculty Guide**

**Total Time: 60 minutes**

**Overview for moderators:**

This faculty development session is being held in an effort to improve endoscopic teaching behaviors in all faculty involved with supervising endoscopy. The content of this session was derived from a qualitative analysis using the observed endoscopic teaching behaviors from the perception of GI fellows. This study identified seven major themes in endoscopic teaching: Teaching, Learning Environment, Communication, Autonomy, Coaching, Professionalism, and Feedback. This session will touch on many aspects of these themes, with a focus on Teaching, Learning Environment, and Communication.

The faculty cohort will observe three videos filmed in an endoscopy suite. These will be simulated interactions to facilitate directed discussion. Through deliberate discussion of observed behaviors and suggestions for improvement, we aim to equip faculty with perspectives on enhancing their own teaching skills.

Equipment Needed:

- Projector/computer AV system with sound capability
- White board/markers to write down comments (optional)

Skills being taught:

- Self-reflection of teaching behaviors that are present in endoscopy
- Verbal and non-verbal communication skills for effective endoscopic teaching
- Delivery of effective feedback

Learning objectives include:

- Examine the impact positive and negative teaching behaviors in endoscopy have on the learning environment
- Apply effective teaching behaviors in endoscopy
- Assess your own endoscopic teaching ability

This guide has an outline for timing of each session. Any text in blue will act a prompt for you.

**INTRODUCTION (3 minutes):**

**Upload Power Point file and start slideshow. Have Videos #1-3 opened and paused.**

**Say:**

We are holding this faculty development session in order to help identify teaching behaviors you can adopt when teaching endoscopy to fellows. We all like to think we are reasonable at teaching endoscopy, and the goal here is not to make everyone a master educator, but to rather take away a few key pointers that you can easily integrate into your precepting the next time you are with a fellow.

**Advance to slide #2. Say:**

The learning objectives for this workshop are:

1. Examine the impact positive and negative teaching behaviors in endoscopy have on the learning environment
2. Apply effective teaching behaviors in endoscopy
3. Assess your own endoscopic teaching ability

The session will be structured around three videos recorded in the endoscopy suite on the consult service with a fellow and attending. While watching each video, take note of any teaching behaviors that you feel are impactful to the fellow’s learning, in either a positive or negative way. We will then reflect as a group and discuss these behaviors further.

**Video Clip #1 (5 minutes)**

**Advance to Slide #3. Say:**

During this clip, observe how successful the attending performs the following:

- Develop an approach to stratify your trainee’s endoscopic skill level
- Identify when a fellow may be struggling with an endoscopic technique
- Understand the balance of autonomy and where a fellow may perceive it to be too little or too much

**Advance to Slide #4. Open and play Video #1.**

**Script Teaching Behavior (Good / Bad)**

*Fellow scoping.*

- **Active involvement in supervision**

***Attending is in background on their phone, not looking at screen***

Fellow encounters clean-based antral ulcer with visible vessel

**Fellow**: “Looks like I found the source.”

- **Discussing endoscopic findings in real time**
- **Expanding knowledge**

*Attending looks up to screen.*

**Attending**: “I agree. **What Forrest classification would you use for that lesion?**

**Fellow**: “Hmmm, 2B?”

- **Creating a supportive learning environment**

**Attending**: “**Close, the letters can be confusing**. 2B is an adherent clot. 2A would be a visible vessel that wasn’t actively bleeding. So how does the Forrest classification help with clinical risk stratification?

**Fellow**: “Well, it believe it helps us better understand rebleeding risk in patients presenting with an upper GI bleed, and therefore help us figure out how to manage them.”

**Attending**: “Correct. How would you like to treat this lesion?”

**Fellow**: “Let’s just burn this.”

**Attending**: “Actually, patients usually have better outcomes in hemostasis when two different modalities are used for peptic ulcer disease. **There was an RCT** that took patients presenting with UGIB secondary to peptic ulcer disease with visible vessels and randomized them to get either epinephrine alone, bipolar cautery alone, or both. The ones that got both had less rebleeding and need for transfusion. Therefore, most of the time we would inject epi and then either clip or burn.”

- **Applying EBM**

**Fellow**: Ok lets inject and burn then.

**Attending**: Actually lets inject and clip. The vessel is in a good position for a clip and the patient is on dual antiplatelet therapy.

- **Discussion of how to perform endoscopic techniques unfamiliar to fellow**

**Fellow**: Ok, I’ve never placed a hemoclip before though.

**Attending**: Haven’t you been on call and done a ton of these by now? **Clip placement is pretty easy let’s just go for it.**

*Voice over: Epinephrine is injected and fellow has threaded the clip through. After about 15 seconds of trying to obtain purchase with the tissue, he accidentally instructs the clip to deploy and it misfires without hitting mucosa.*

- **Assists fellow when unable to complete endoscopic tasks**

**Fellow**: “Oh crap, I accidentally fired the clip.”

**Attending**: “That’s okay, **you only wasted $200 of taxpayer money**. Go ahead and try again.”

- **Condescending comment**
- **Creating a supportive learning environment**

**Fellow**: “I’m not sure what I’m doing wrong.”

Attending: “**That’s okay, this time you’ll get it**.”

- **Demonstrates understanding of fellow’s perceived skill level**

**Fellow**: “Okay.”

- **Guides fellow through performing difficult tasks commiserate with their skill level**

*Fast forward, fellow misfires again.*

**Attending**: OK let me show you how it’s done.

**Attending grabs scope from fellow.**

- **Demonstrates endoscopic techniques unfamiliar to fellow**

*Attending asks for another clip and* ***places it without discussing what he is doing****. Fellow is watching the screen, arms crossed. Hemostasis is achieved.*

**Attending**: “See, that’s how you do it.”

**Fellow**: “Oh, okay.”

**INTERMISSION (10 minutes):**

**Ask the audience the following prompts. Write down the comments (optional).**

1. How did this encounter resonate with any of you? *Take 2-3 responses, directly ask physicians if no volunteers.*
2. What behaviors did you notice that might have enhanced the fellow’s education? *Write these down*
3. What behaviors did you notice that might have harmed the fellow’s education? *Write these down*

**A representative selection of behaviors is included here:**

| DIRECT TEACHING  **Discussed endoscopic findings in real time in regards to Forrest Classification, which also helped expand knowledge.**  **Applied EBM in regards to dual therapy.**  **Didn’t explain how to perform endoscopic techniques unfamiliar to fellow (clip placement)** |
| --- |
| LEARNING ENVIRONMENT  **Poor supervision; on phone in beginning of encounter**  **Condescending comment (wasting money on misfired clip)** |
| COMMUNICATION  **Didn’t communicate on how to place the clip. Just did it.** |
| AUTONOMY  **Gave too much autonomy when fellow unable to perform task, fellow unsupported.** |

**Pull up the power point file.**

**Advance to Slide #5.**

**Video Clip #2 (5 minutes)**

**Say:** This will be the same clinical case, but with a slightly different teaching approach from the attending.

During this clip, please observe for the following:

- Effective endoscopic teaching techniques to apply in real time when you encounter a fellow who is struggling
- Differentiate verbal and non-verbal methods of procedural teaching

**Advance to Slide #6. Play Video #2.**

**Script Teaching Behavior (Good / Bad)**

*Fellow has just started scoping. The* ***attending is standing directly to the left of him, facing the monitor and intermittently looking at the fellow’s hands****.*

- **Active involvement in supervision**

*Fellow encounters an antral ulcer with visible vessel.*

- **Discussing endoscopic findings in real time**

**Attending**: “**What do you see here?”**

**Fellow**: “Looks like a clean-based ulcer and a visible vessel. This is probably what caused the melena.”

**Attending**: “Most likely, but let’s clear the duodenum before dealing with it.”

*Voice over: Fellow gets into the duodenal bulb, but has issues getting into the 2^nd^ portion of the duodenum.*

- **Allows fellow to perform procedure at their skill level without interruption**
- **Demonstrates understanding of fellow’s perceived skill level**
- **Demonstrates patience**

**Attending**: “Let me get you into the 2^nd^ portion real quick.”

**Fellow**: “I can do it, for some reason her sweep is tortuous.”

*Fellow tries for a few more seconds.*

**Attending**: “Yeah, that can happen sometimes, here let me see if I can take a feel”

***Attending takes the scope.***

**Attending**: “**The key move here is to drop your left arm to the right, just like this. This will usually place the scope in the right position and get you into the 2^nd^ portion**.” Attending gets into the 2^nd^ portion. “**You see?”**

- **Discussion of how to perform endoscopic techniques unfamiliar to fellow**
- **Uses effective verbal communication skills**

**Fellow**: “Oh, cool. Yes I see. I’ll make sure to do that next time.”

*Attending withdrawals back into the stomach. Hands the scope back to the fellow.*

**Attending**: “Okay, let’s inject and clip this lesion now.”

- **Expanding knowledge**
- **Creating a supportive learning environment**

**Fellow**: “Oh, I was just thinking we could burn it.”

**Attending**: “Let’s inject and clip instead.”

**Fellow**: “Okay.”

*Fellow has injected epi and has now fed the hemoclip through the scope.*

**Fellow**: “So, I haven’t had a chance to place one of these yet.”

**Attending**: “Ok no problem. Lets feed the clip so you see it come all the way out of your scope.”

- **Discussion of how to perform endoscopic techniques unfamiliar to fellow**
- **Guides fellow through difficult tasks commiserate with their skill level**

**Fellow**: “Like this?”

**Attending**: “Yes. Now you can ask the tech to open and close the clip. Go ahead.”

**Fellow**: “Okay. Can you open the clip? Okay, can you close it?”

**Attending**: “**The important thing here is to make sure you specify between just simply closing the clip and actually deploying it. You can practice grabbing the tissue and closing, and then tell the tech to ‘deploy’ once you find a satisfactory position**.”

**Fellow**: “Okay, makes sense.”

**Attending**: “Ok let see if you can clip this thing.”

*Fellow pushes the clip out and has difficulty with positioning. Camera angle shows attending looking directly at his hands.*

**Fellow**: “I can’t seem to get the right angle.”

- **Guides fellow through difficult tasks commiserate with their skill level**

**Attending**: “**To gain more control, I want you to pull back on the clip all the way to the tip of the scope so you can see just the prongs. Most of your movement towards the lesion should be with the scope itself. The less distance your clip is from your scope, the more control you’ll have**.”

*Fellow starts practicing better positioning.*

**Attending**: “Yes just like that.”

*Fellow gets good positioning on the lesion.*

**Attending**: “Deploy there.”

**Fellow**: “Ok, deploy.”

*Clip is successfully placed.*

**Attending**: “Good job.”

**INTERMISSION (10 minutes):**

**Ask the audience the following prompts. Write the comments (optional).**

*Ask the audience:*

1. What was different about this encounter? *Take 2-3 responses, directly ask physicians if no volunteers.*
2. What are some ways you can gauge a fellow’s skill level? *Take 2-3 responses, directly ask physicians if no volunteers.*
3. What different behaviors (positive or negative) did you notice here compared to the prior clip? *Write these down.*

| DIRECT TEACHING  **Discussing endoscopic findings in real time**  **Discussed how to perform endoscopic techniques unfamiliar to fellow (duodenal intubation, hemoclip placement)**  **Guides fellow through difficult tasks commiserate with their skill level (obtaining better purchase with hemoclip)**  **Didn’t explain why burning the lesion wasn’t the right move** |
| --- |
| LEARNING ENVIRONMENT  **Active involvement in supervision by standing next to fellow and looking at hands**  **Didn’t value fellow’s difference in opinion on therapeutic maneuver** |
| COMMUNICATION  **Uses effective verbal communication skills in teaching duodenal intubation.** |
| AUTONOMY  **Didn’t allow fellow to perform procedure at their skill level without interruption in regards to duodenal intubation. Didn’t demonstrate understanding of fellow’s perceived skill level** |

**Once you are satisfied with the responses, move to the next video.**

**Video Clip #3 (5 minutes)**

**Pull up the power point file.**

**Advance to Slide #7. Say:**

During this last clip, please observe for behaviors related to providing effective feedback.

**Advance to Slide #8. Play Video #3.**

**Script Teaching Behavior (Good / Bad)**

*Fellow has just finished writing the endoscopy report.*

- **Reviews endoscopy report**

**Fellow**: “Ok the report is ready for you to sign.”

***Attending is reading the report****.*

**Attending**: “Hmm, you forgot to mention all the blood we saw in the stomach

and how we needed to spend some time cleaning. This is important to note

because the team should expect to see some melena even though the source

of bleeding is treated. Also, I would mention that H.pylori testing should be

performed since we didn’t biopsy during the procedure.”

**Fellow**: “Ok sounds good.”

**Attending** “You think we can advance his diet and discharge him?”

**Fellow**: “Yeah, I mean we treated the lesion and had hemostasis so he should be set.”

- **Expands knowledge**

**Attending**: “I wouldn’t say that the patient could be discharged. We should check his hemoglobin tomorrow. **An ulcer with visible vessel is high-risk stigmata of bleeding. Theoretically, based on the literature we should also consider keeping this patient on IV PPI for 72 hours.**”

**Fellow**: “Oh I see, that makes sense.”

**Attending**: **“Is it okay if I give you some feedback on how that procedure went?”**

- **Provides formal feedback**

**Fellow**: “Yes, of course.”

**Attending**: “How did you think it went?”

**Fellow**: “Well, I think it took me a while to get the clip right, but am pretty happy with how it went since I didn’t need to waste one or anything.”

**Attending**: “Anything you wanted to work on going forward?”

**Fellow**: “Well, I guess I wanted to incorporate that duodenal intubation technique you showed me.”

**Attending**: “Ok sounds good, I’ll make sure to keep an eye out for that during the next EGD. Anything else?”

**Fellow**: “Can’t think of anything.Thanks for the feedback.”

**Attending**: “No problem.”

**INTERMISSION (10 minutes):**

**Ask the audience:**

1. What did you notice in this clip? Does this resonate with your practice? *Take 2-3 responses, directly ask physicians if no volunteers.*
2. In what ways can you incorporate more feedback in your encounters?

**Advance to Slide #9. Say:**

The content of this session was derived from a qualitative analysis using the observed endoscopic teaching behaviors from the perception of GI fellows. This study identified seven major themes in endoscopic teaching: Teaching, Learning Environment, Communication, Autonomy, Coaching, Professionalism, and Feedback. This session will touched on many aspects of these themes. Teaching in endoscopy is multifaceted. There are several domains in which we can improve our ability to teach. All of us have an area we could improve upon.

**Conclusion (2 minutes):**

**Advance to Slide #10. Say*:***

To recap, through this session we were able to discuss and improve our endoscopic teaching in the following ways:

- There are many different teaching behaviors that are involved in an endoscopy encounter
- There are ways to demonstrate unfamiliar endoscopic techniques that require both verbal and non-verbal instruction.
  - If you take the scope, teach!
- Diagnosing your fellow’s learning style is important.
  - For instance, too much verbal instruction may be ineffective for first years due to their cognitive while a third year fellow should be able to multitask.
  - Incorporating EBM may not be appropriate during an endoscopy, but consider adding supplementing the fellow’s knowledge base pre- or post-procedure
- Providing formal feedback is a key step in improving endoscopic learning.

**Advance to Slide #11. Say*:***

Thank you for your time and participation. Any questions?
