# Supplementary material for: Faculty Development Workshop for Endoscopic Teaching Techniques
Source: MedEdPORTAL. 2020 Sep 23;16:10960. doi: 10.15766/mep_2374-8265.10960 (PMC7511062; doi:10.15766/mep_2374-8265.10960)
Supplement: Supplementary file 1 — Video 1.mp4Video 2.mp4Video 3.mp4Facilitator Guide.docxWorkshop Slides.pptxPre- and Postworkshop Survey.docx [file mep_2374-8265.10960-s001.zip › E. Workshop Slides.pptx]

## Slide 1
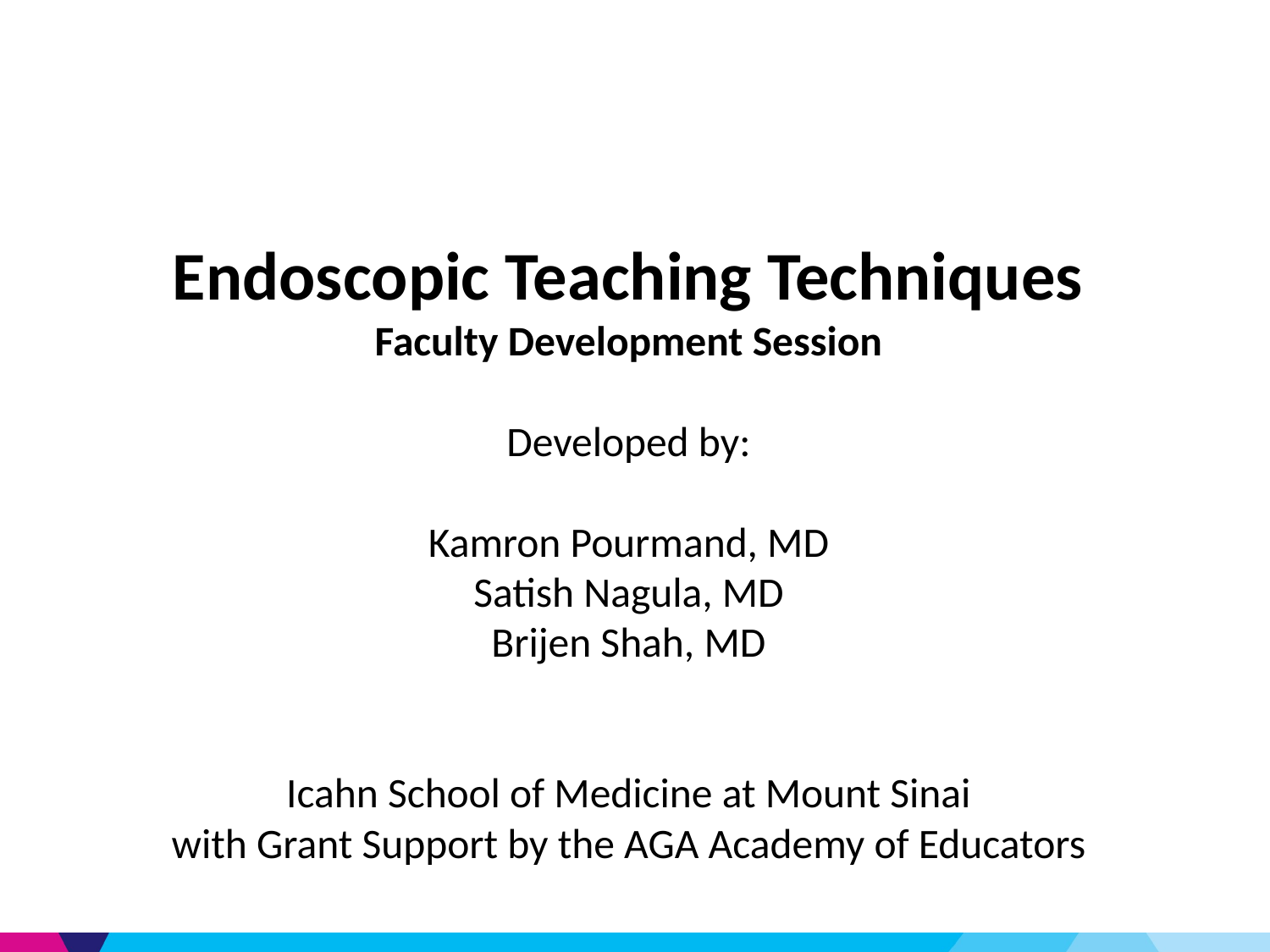

Endoscopic Teaching Techniques
Faculty Development Session
Developed by:
Kamron Pourmand, MD
Satish Nagula, MD
Brijen Shah, MD
Icahn School of Medicine at Mount Sinai
with Grant Support by the AGA Academy of Educators

## Slide 2
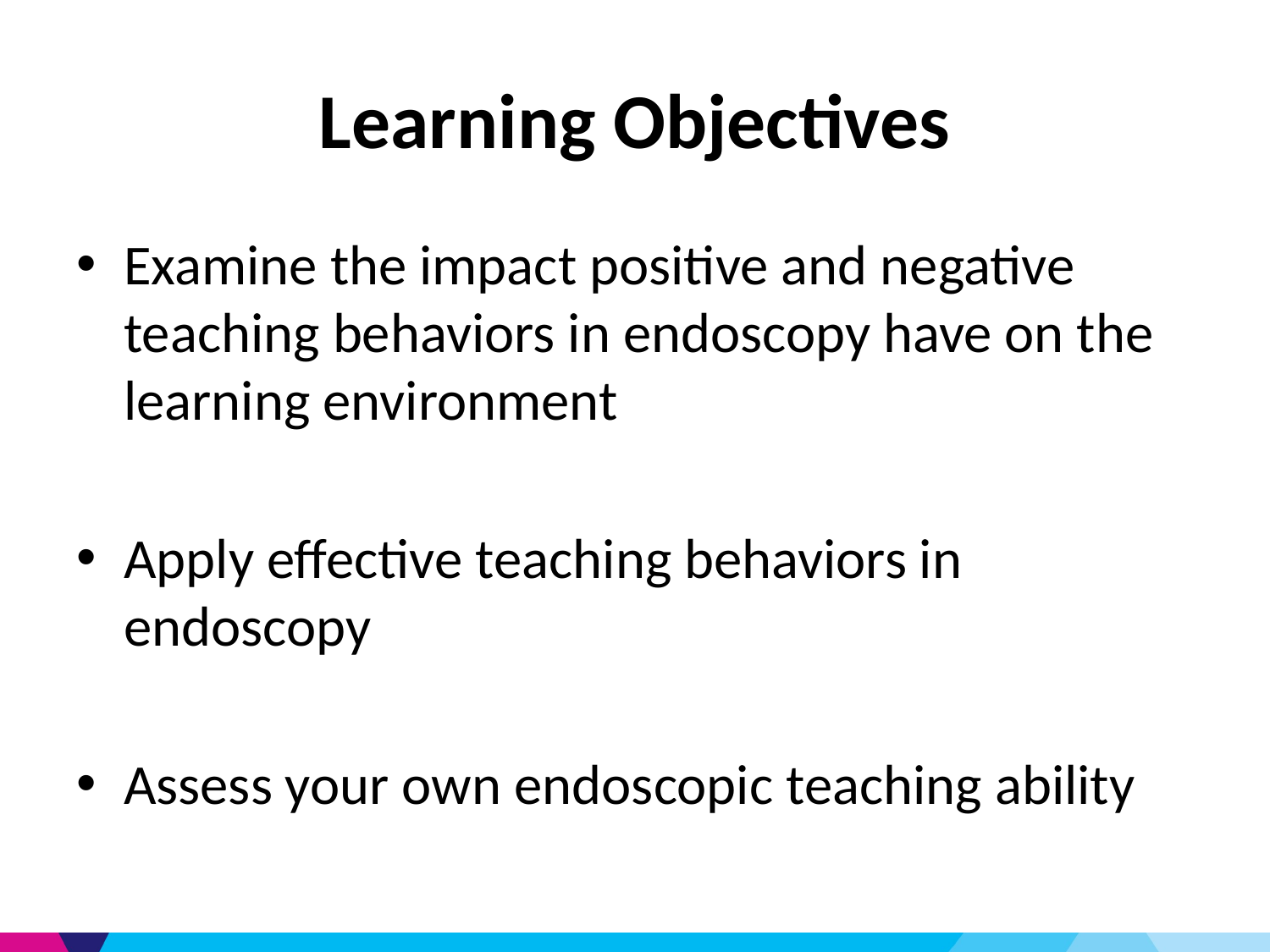

# Learning Objectives
Examine the impact positive and negative teaching behaviors in endoscopy have on the learning environment
Apply effective teaching behaviors in endoscopy
Assess your own endoscopic teaching ability

## Slide 3
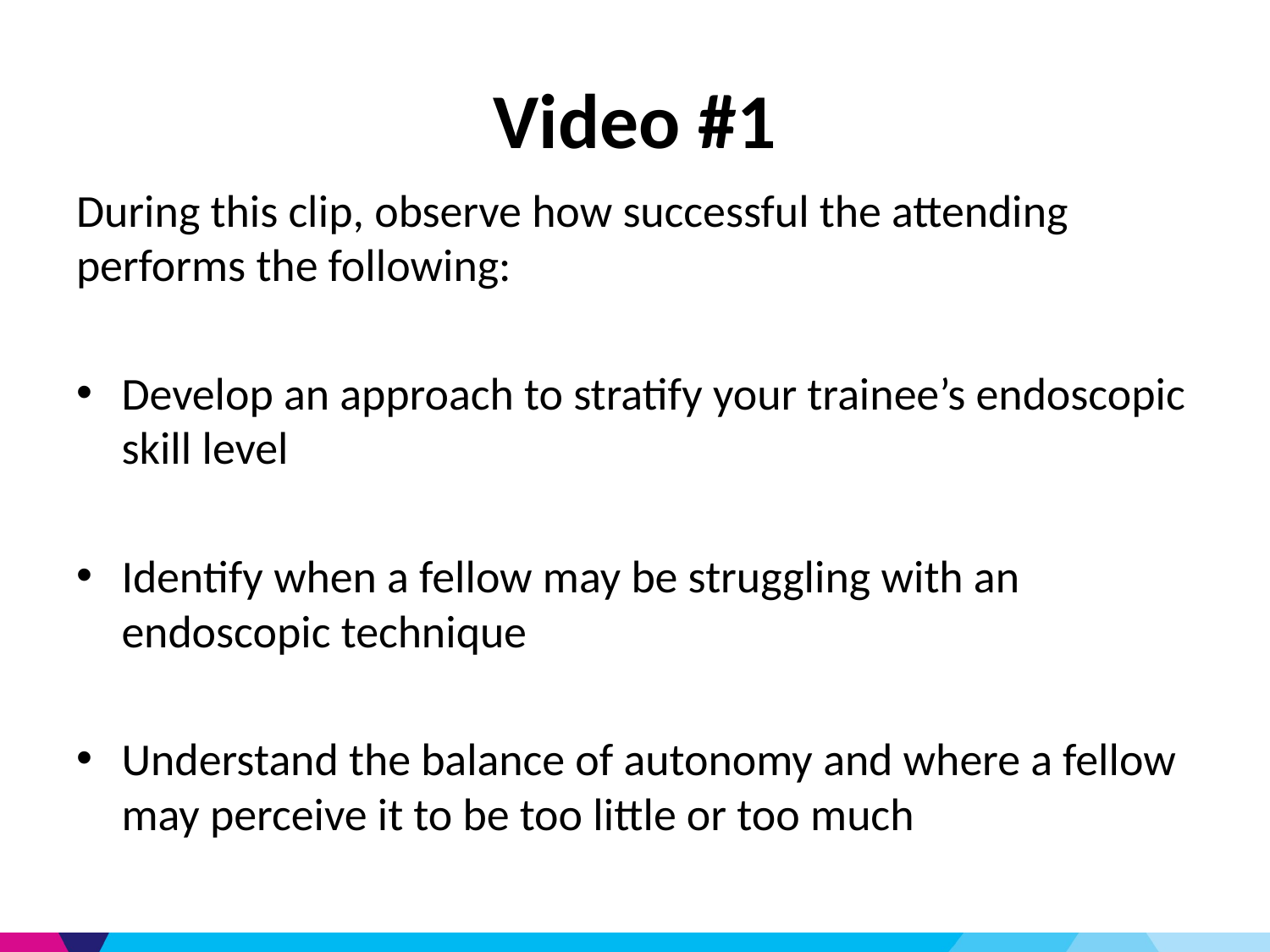

# Video #1
During this clip, observe how successful the attending performs the following:
Develop an approach to stratify your trainee’s endoscopic skill level
Identify when a fellow may be struggling with an endoscopic technique
Understand the balance of autonomy and where a fellow may perceive it to be too little or too much

## Slide 4
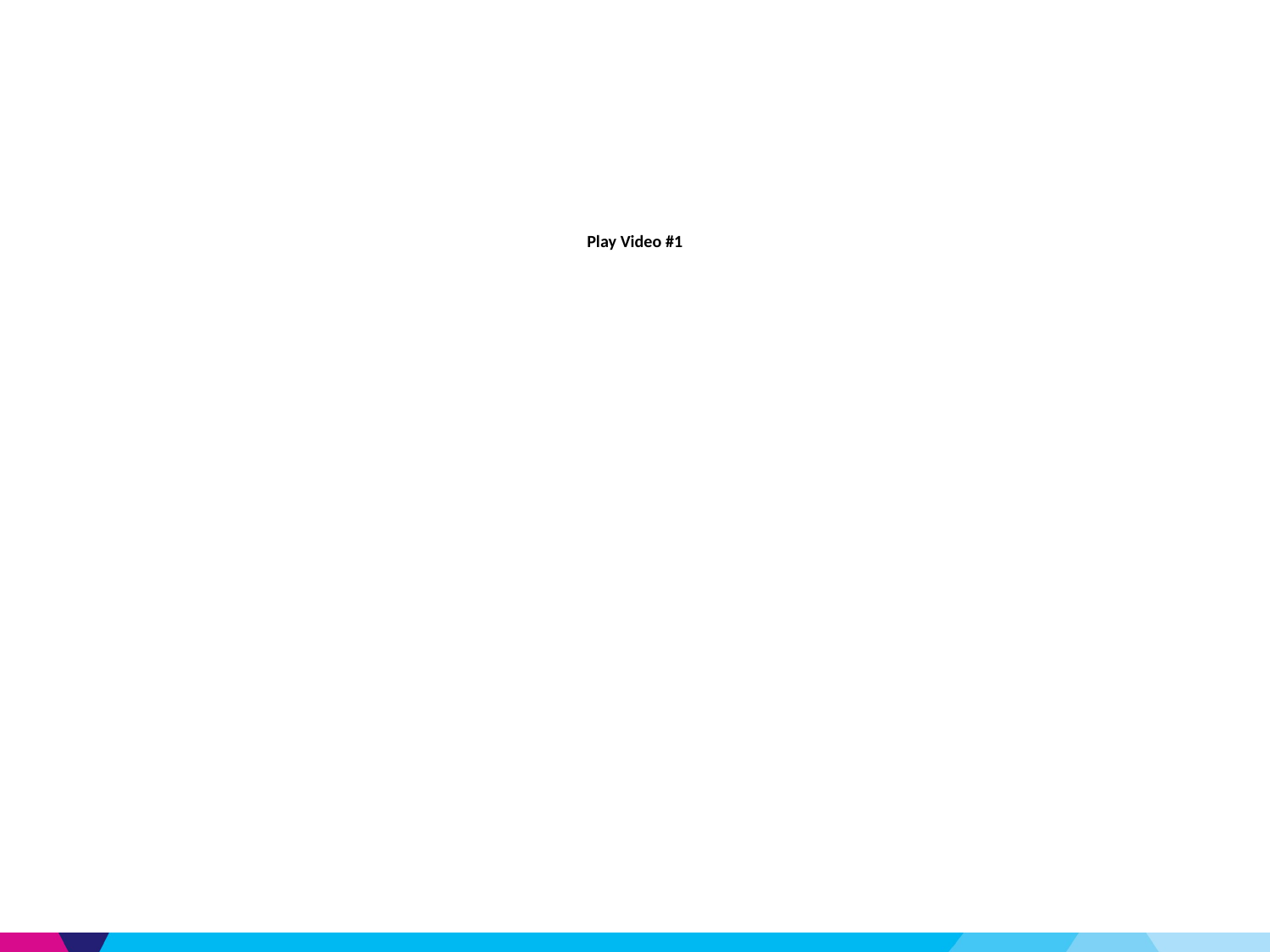

# Play Video #1

## Slide 5
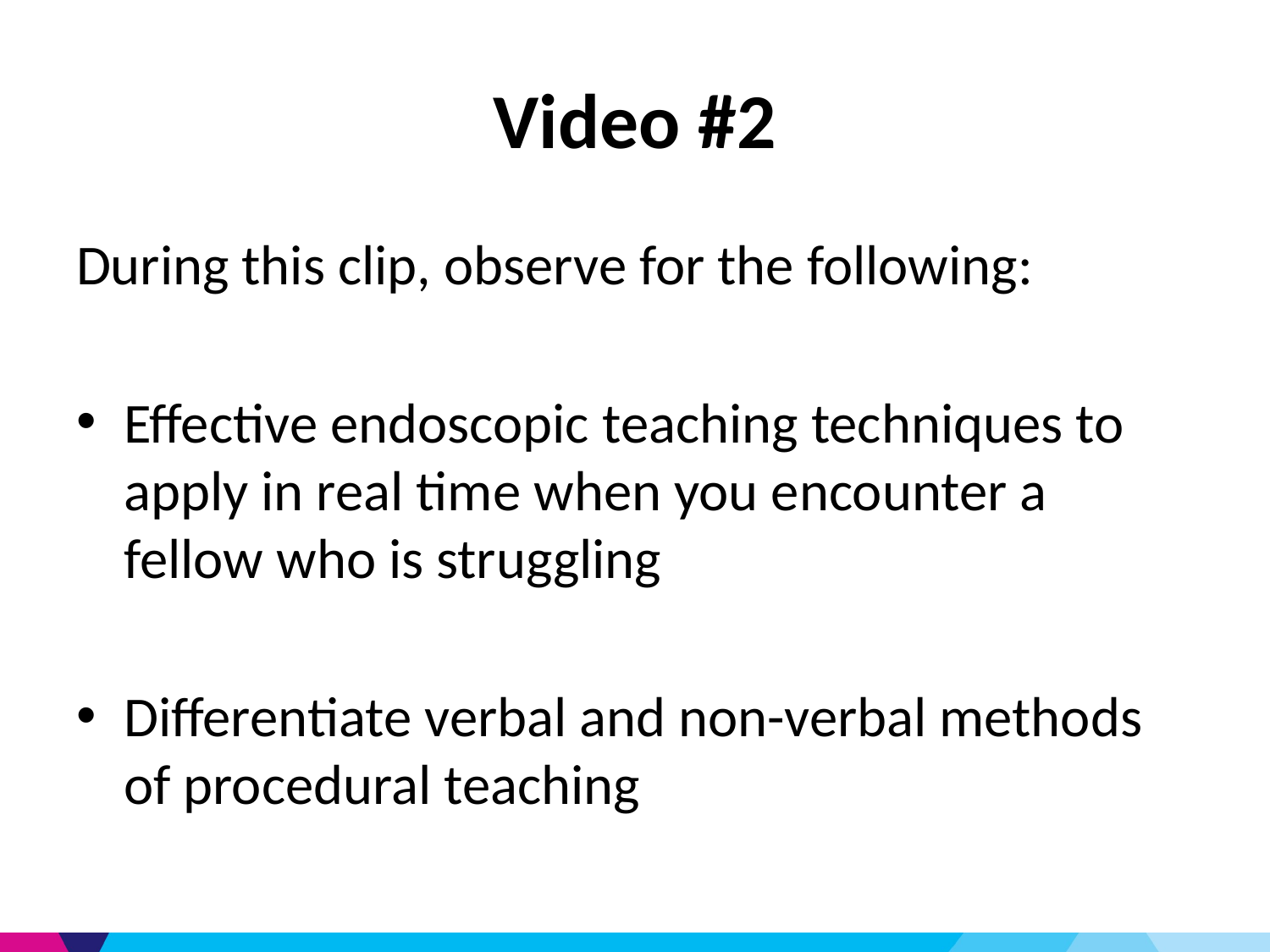

# Video #2
During this clip, observe for the following:
Effective endoscopic teaching techniques to apply in real time when you encounter a fellow who is struggling
Differentiate verbal and non-verbal methods of procedural teaching

## Slide 6
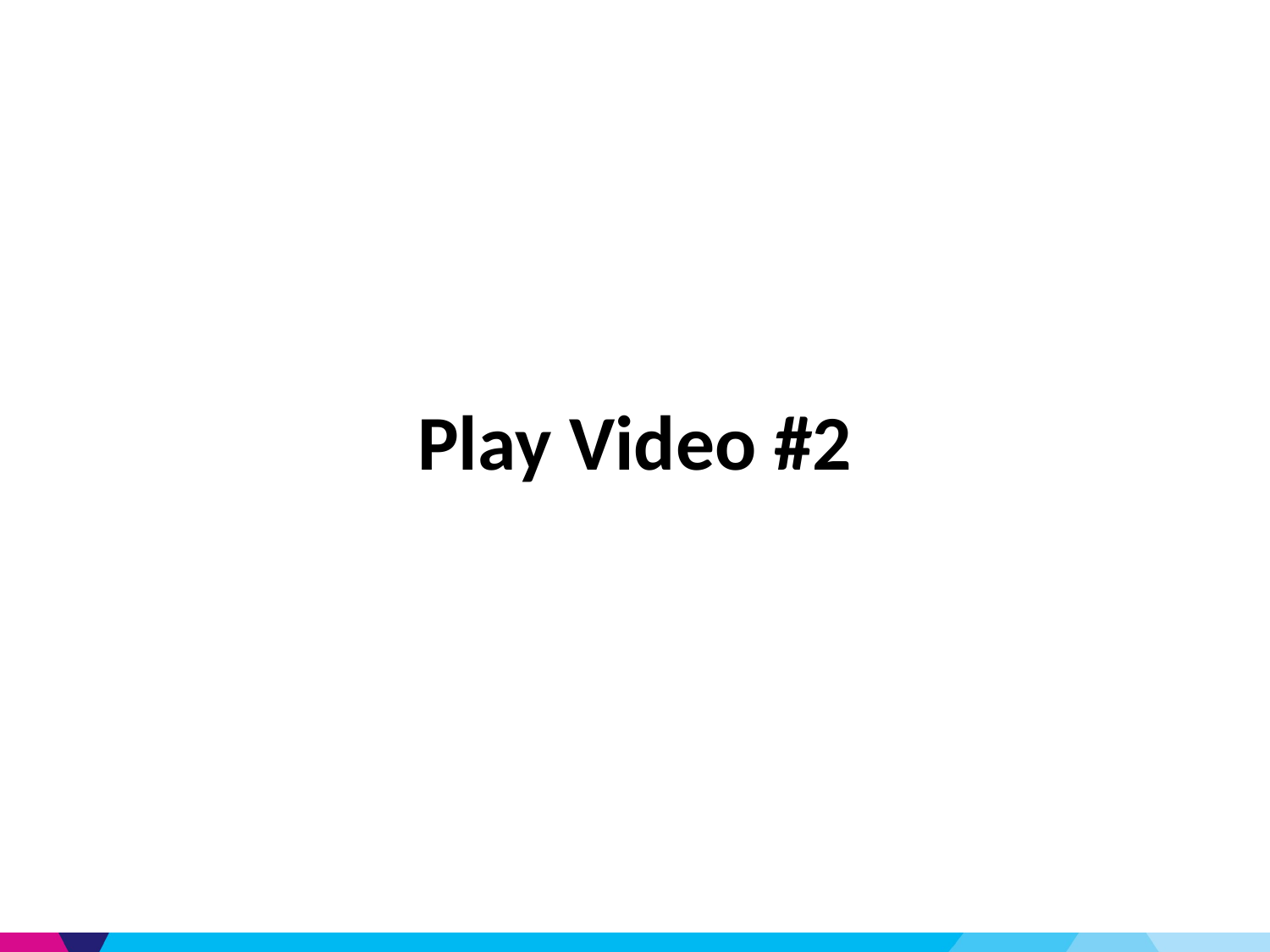

# Play Video #2

## Slide 7
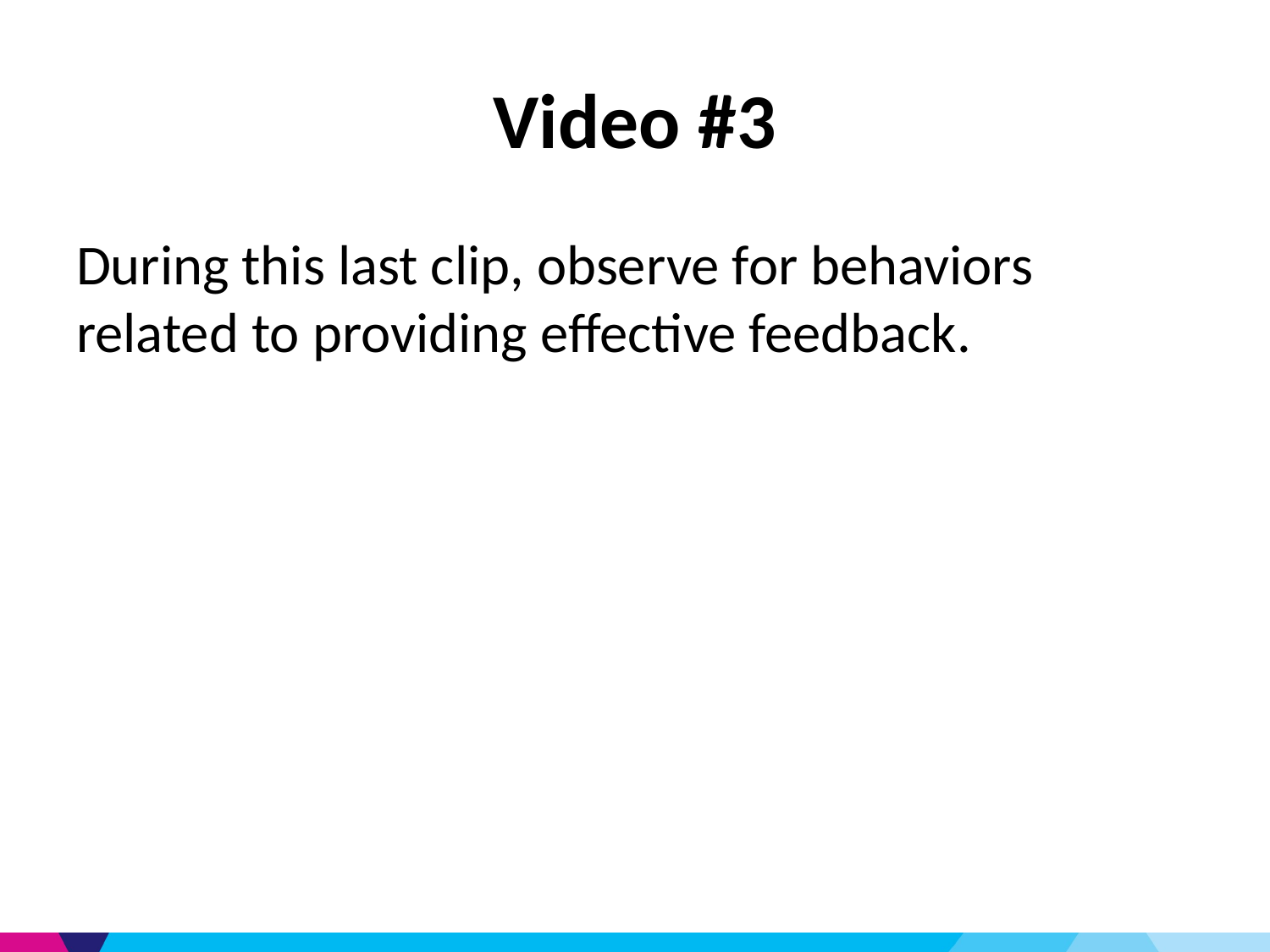

# Video #3
During this last clip, observe for behaviors related to providing effective feedback.

## Slide 8
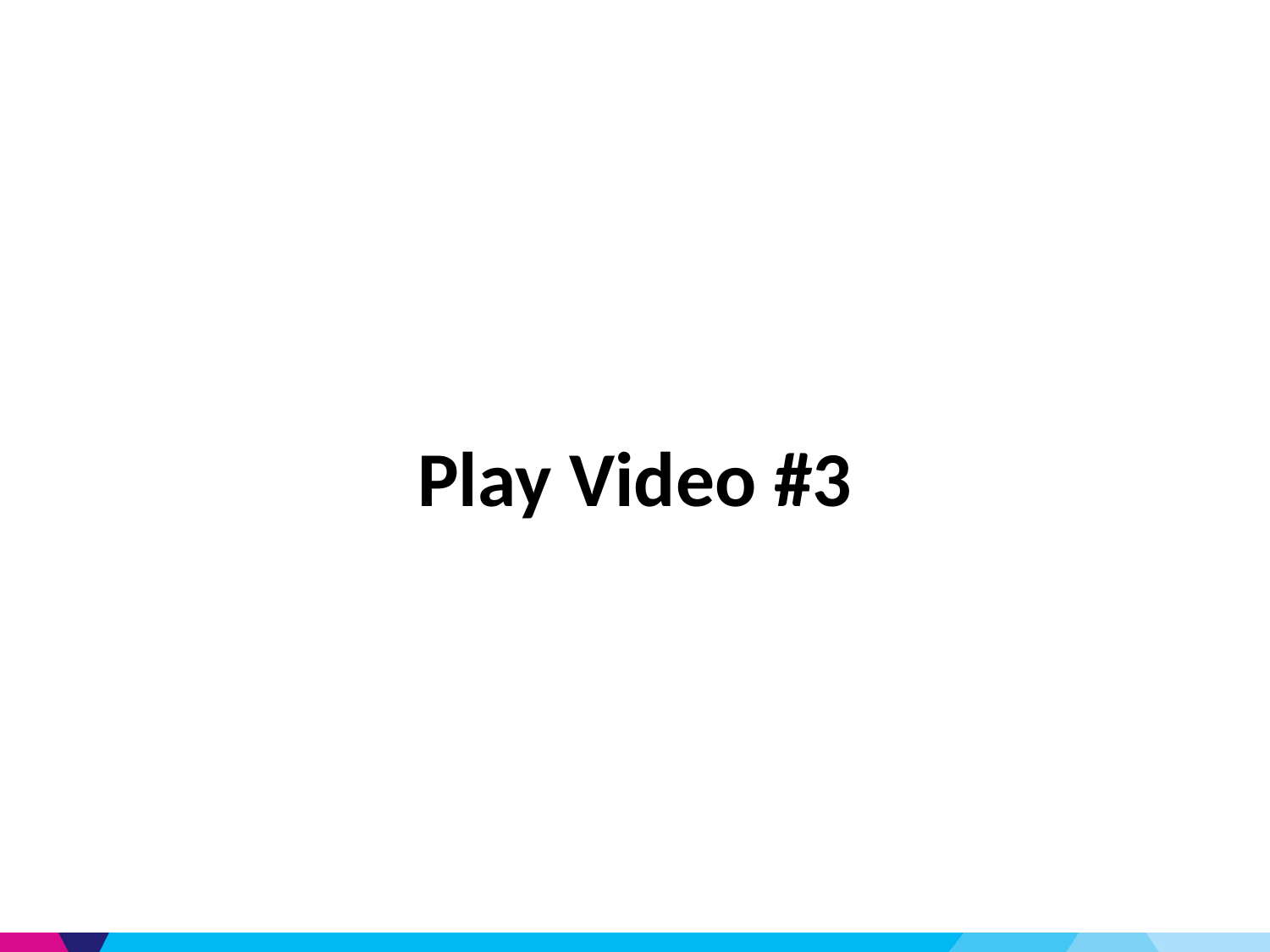

# Play Video #3

## Slide 9
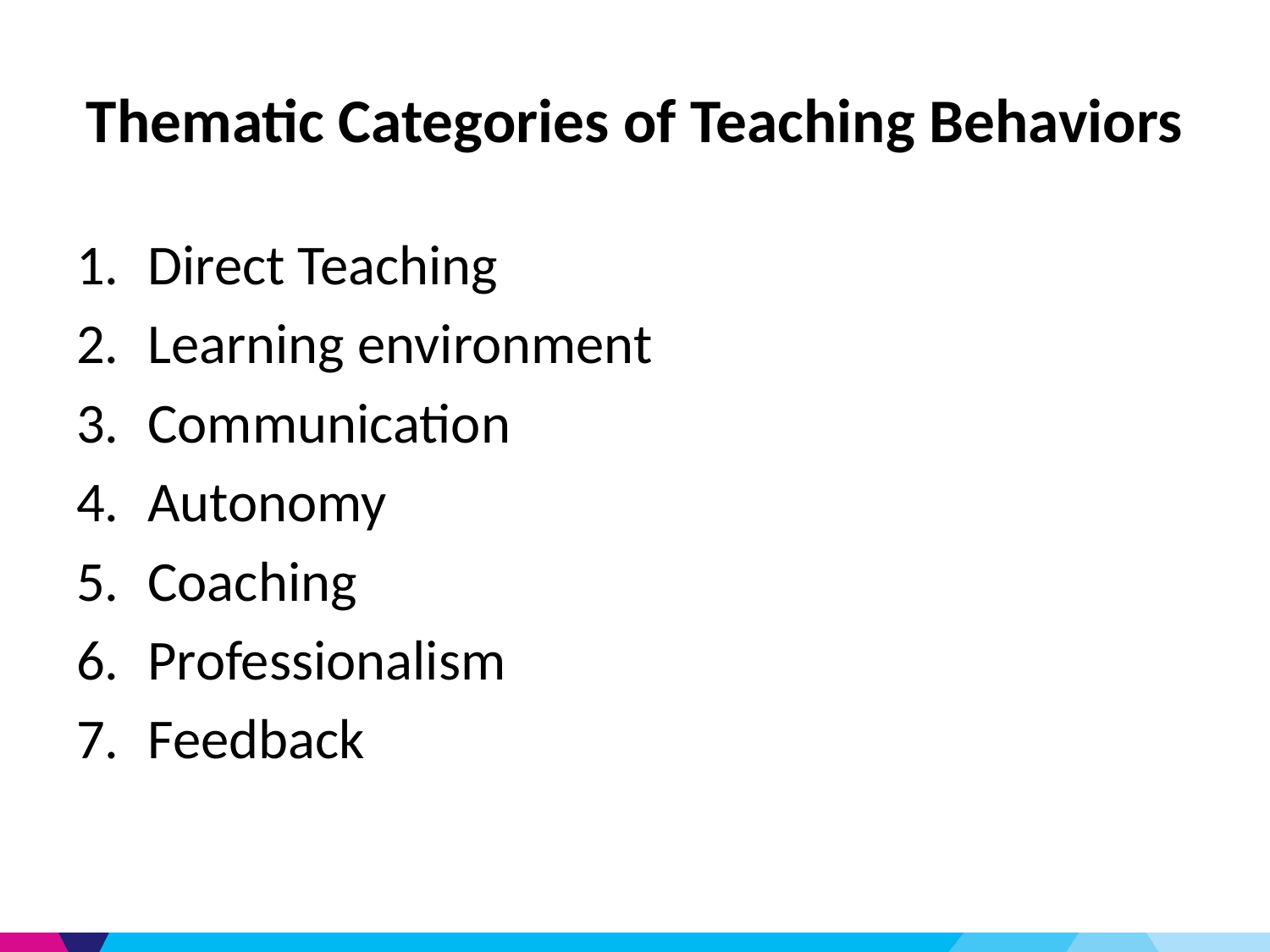

# Thematic Categories of Teaching Behaviors
Direct Teaching
Learning environment
Communication
Autonomy
Coaching
Professionalism
Feedback

## Slide 10
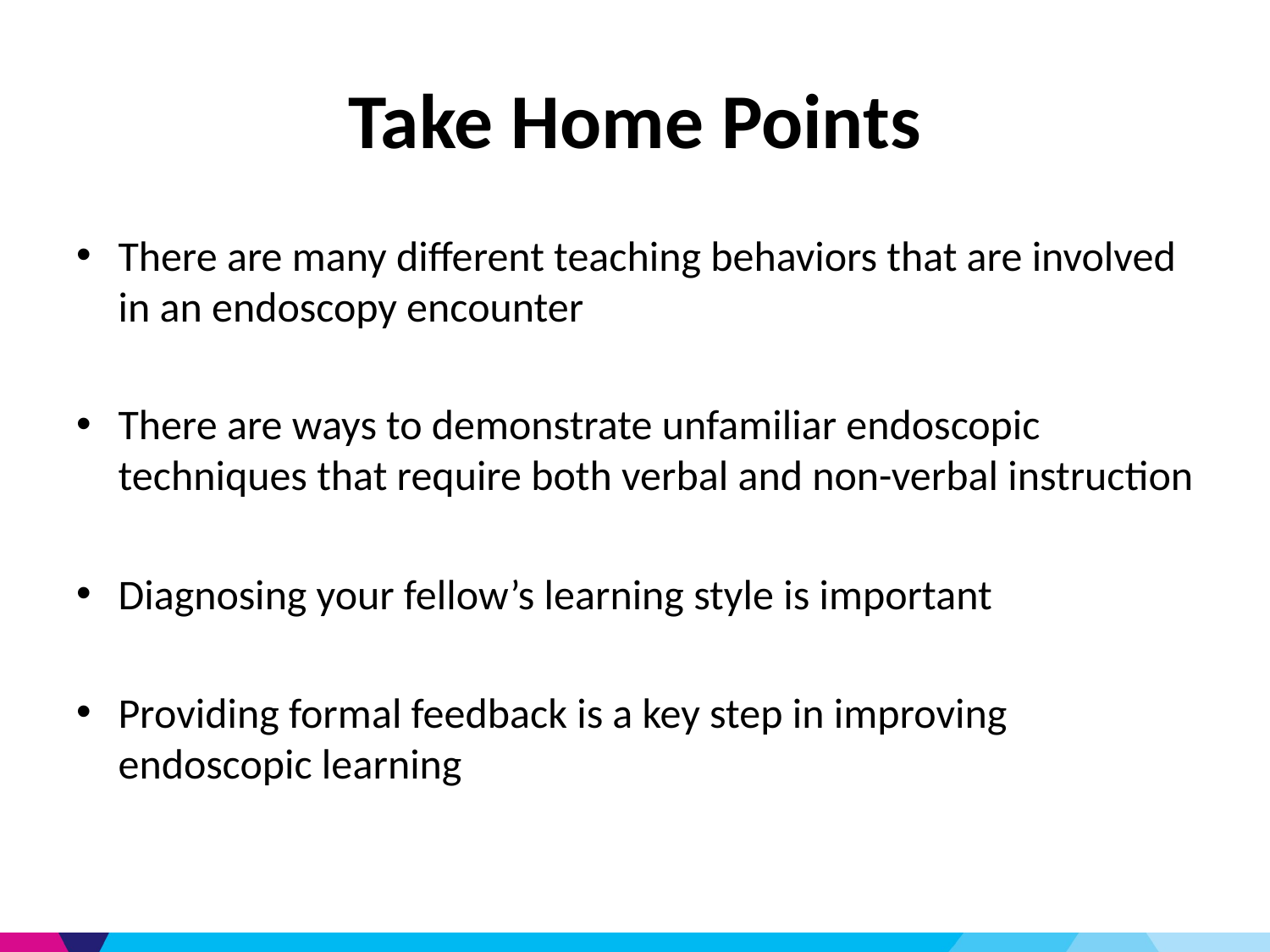

# Take Home Points
There are many different teaching behaviors that are involved in an endoscopy encounter
There are ways to demonstrate unfamiliar endoscopic techniques that require both verbal and non-verbal instruction
Diagnosing your fellow’s learning style is important
Providing formal feedback is a key step in improving endoscopic learning

## Slide 11
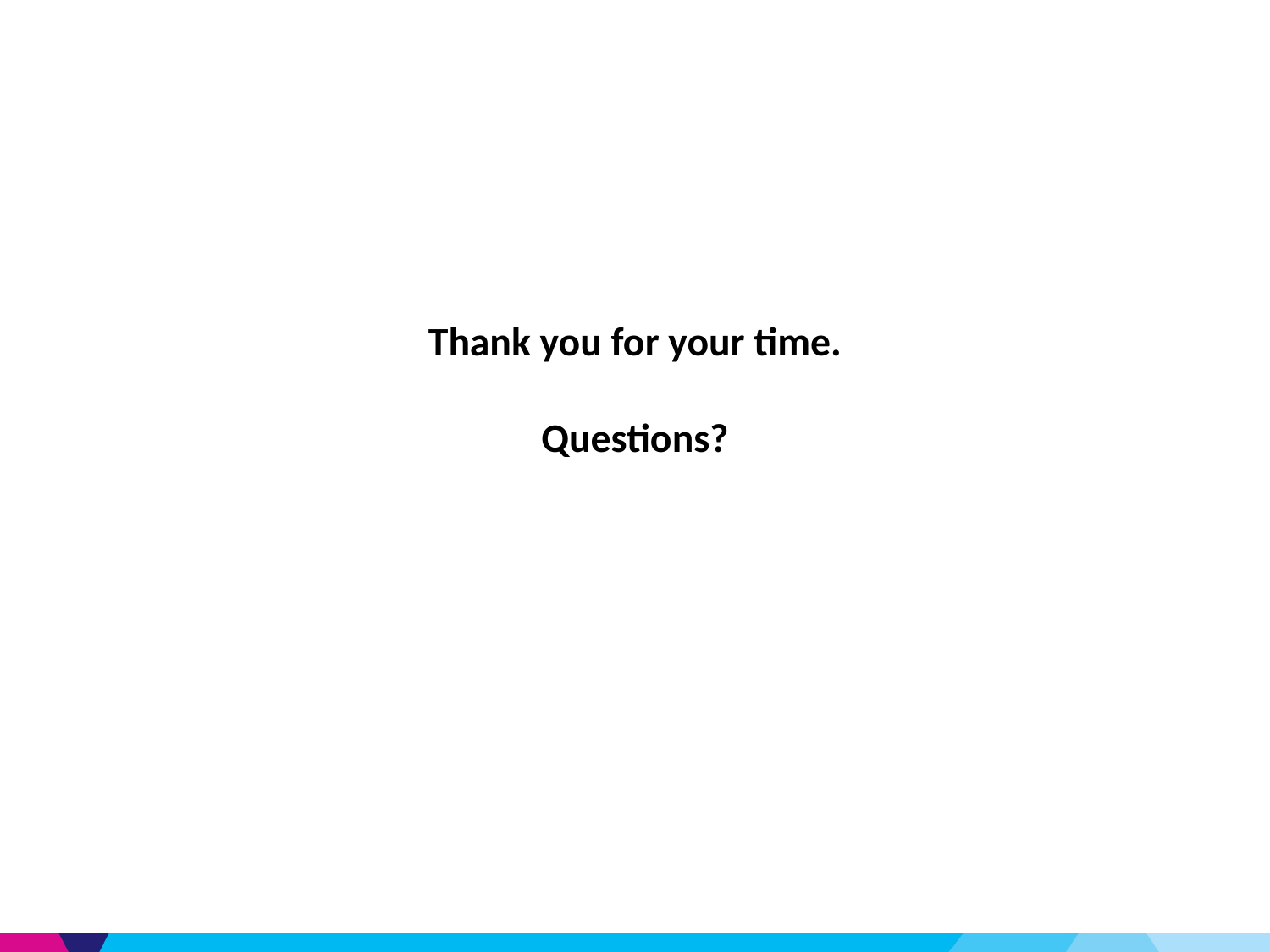

# Thank you for your time. Questions?
