# Supplementary material for: Faculty Development Workshop for Endoscopic Teaching Techniques
Source: MedEdPORTAL. 2020 Sep 23;16:10960. doi: 10.15766/mep_2374-8265.10960 (PMC7511062; doi:10.15766/mep_2374-8265.10960)
Supplement: Supplementary file 1 — Video 1.mp4Video 2.mp4Video 3.mp4Facilitator Guide.docxWorkshop Slides.pptxPre- and Postworkshop Survey.docx [file mep_2374-8265.10960-s001.zip › F. Pre- and Postworkshop Survey.docx]

**How many years have you taught endoscopy?**

| **<5 years** | **5-10 years** | **>10 years** |
| --- | --- | --- |

**Pre-Session Survey**

1. Please self-assess your ability to teach endoscopy.

Very Ineffective Very Effective

| **1** | **2** | **3** | **4** | **5** |
| --- | --- | --- | --- | --- |

1. How often do you provide feedback to your trainees?

Never After each encounter

| **1** | **2** | **3** | **4** | **5** |
| --- | --- | --- | --- | --- |

**Post-Session Survey**

1. Please re-assess your ability to teach endoscopy

Very Ineffective Very Effective

| **1** | **2** | **3** | **4** | **5** |
| --- | --- | --- | --- | --- |

1. I am more aware of the teaching behaviors that I demonstrate and how they impact trainees.

Strongly Disagree Strongly Agree

| **1** | **2** | **3** | **4** | **5** |
| --- | --- | --- | --- | --- |

1. This session helped me discover or reinforce effected teaching behaviors

Strongly Disagree Strongly Agree

| **1** | **2** | **3** | **4** | **5** |
| --- | --- | --- | --- | --- |

1. I was able to discuss/reflect on the impact I have in the learning environment

Strongly Disagree Strongly Agree

| **1** | **2** | **3** | **4** | **5** |
| --- | --- | --- | --- | --- |

1. How likely are you to change your endoscopic teaching practices as a result of this session?

Very Unlikely Very Likely

| **1** | **2** | **3** | **4** | **5** |
| --- | --- | --- | --- | --- |

**If you intend to change your teaching practices as a result of this session, what is it that you intend to do?**

_____________________________________________________________________________________________

_____________________________________________________________________________________________
